# Supplementary material for: Chromothripsis during telomere crisis is independent of NHEJ, and consistent with a replicative origin
Source: Genome Res. 2019 May;29(5):737–49. doi: 10.1101/gr.240705.118 (PMC6499312; doi:10.1101/gr.240705.118)
Supplement: Supplemental Material [file supp_gr.240705.118_Supplemental_file_1.zip › contigs/annotated_contigs/DB93/contig.3.DB93_length_759_mean_cov_7.70750988142.docx]

**DB93_length_759_mean_cov_7.70750988142**

```
ATTTTAAATAGGTTAGAGATGTTTTTAAATCCTATTTGAAACTCATTAACAAAACCATTCCTGTGCAATAGCCAAATTTTTATGAGGAG
 >chr3:7184509-7184658 + E=8e-75
AAAAGAATAATAAGATGACAATATGGTCCTGTGATACAGATTAAAAATATTTTAGTT|TTC|CCAAGACTCTTCCCCTTGCCACTTGCA
                                                          >chr3:7495032-7495389 + E=6e-
ATGACAGTGGCAAACCCTAGGTTGTTTTGTCTGGGCTTCTGATGGTCCGGCTATAAACTGGAATTTTCACAATCTCCTCCTCAGGTTTG
203
ATTAATTTGCTAGAGCAACTCACAAAACTCAGTAAAACACTTACTTAAATTTATTACAAAAGGTATTTTAAAGGACACAAATAAACCTC

CAGATGAAGAGATCCTAGGGCAAAGTCTGAAAGGGTCCTGAGAGTAGGAGTTTCTGTCCTCAGGGATTTGGGGTGTGCTGGCCTCCCCT

GCACAAAGATGAGTTCTTGCTCACTTTCCTGTCAGCCTCCATGTGTTCAACTCTGAACCT|AAAT|ATTTGATGCTTGAATTGGCCAGT
                                                                 >chr3:4491252-4491504
TGAGATTTATAAGCACATTTGACCCCTTAGTGGAACTAGAATGTGTCAGAATGTGAGCAAACAGTGCTATTTAATTCGTTTAGCTAGAT
- E=9e-140
TCAGGACAAAGGACAAAGATTGATTATGTAATCTCACACGTACAGAATACTATTTCCCAGAGTCATTTGTGATACTTGGGGAAATGTAT

TTCCTTCAAGAACAGTCACGTTCTCAAAAGCTAATCTTGTTCAGTCTGCAT
```
